# Impact of low-density lipoprotein cholesterol on progression of aortic valve sclerosis and stenosis

**DOI:** 10.3389/fcvm.2023.1171703

**Published:** 2023-07-17

**Authors:** Jeong Hun Seo, Kang Hee Kim, Kwang Jin Chun, Bong-Ki Lee, Byung-Ryul Cho, Dong Ryeol Ryu

**Affiliations:** Division of Cardiology, Department of Internal Medicine, Kangwon National University Hospital, Kangwon National University School of Medicine, Chuncheon-si, Republic of Korea

**Keywords:** LDL cholesterol, aortic valve sclerosis, peak aortic jet velocity, aortic stenosis, echocardiogaphy

## Abstract

**Background:**

Little research has been assessed atherosclerotic risk factors at various stages of calcific aortic valve disease. This study sought to determine risk factors of patients with aortic valve sclerosis (AVS) and mild to moderate aortic stenosis (AS).

**Methods:**

The study included 1,007 patients diagnosed with AVS or mild to moderate AS according to echocardiographic criteria. Patients were identified as a rapid progression group if the annualized difference in peak aortic jet velocity (Vmax) between two echocardiographic examinations was >0.08 m/s/yr in AVS and >0.3 m/s/yr in AS, respectively. We used multivariable logistic regression analyses to assess the factors associated with rapid disease progression or progression to severe AS.

**Results:**

Among 526 AVS patients, higher LDL-C level (odds ratio [OR] 1.22/per 25 mg/dl higher LDL-C, 95% confidence interval [CI] 1.05–1.43) was significantly associated with rapid disease progression. Compared to patients with LDL-C level <70 mg/dl, the adjusted OR for rapid progression were 1.32, 2.15, and 2.98 for those with LDL-C level of 70–95 mg/dl, 95–120 mg/dl, and ≥120 mg/dl, respectively. Among 481 mild to moderate AS patients, the baseline Vmax (OR 1.79/per 0.5 m/s higher Vmax, 95% CI 1.18–2.70) was associated with rapid progression. Compared to patients with Vmax 2.0–2.5 m/s, the adjusted OR for rapid progression were 2.47, 2.78, and 3.49 for those with Vmax of 2.5–3.0 m/s, 3.0–3.5 m/s, and 3.5–4.0 m/s, respectively. LDL-C and baseline Vmax values were independently associated with progression to severe AS.

**Conclusion:**

Atherosclerotic risk factors such as LDL-C were significantly associated with the rapid progression in AVS and baseline Vmax was important in the stage of mild to moderate AS.

## Introduction

Calcific aortic valve (AoV) disease is a progressive condition from aortic valve sclerosis (AVS), mild leaflet thickening without valve obstruction, to severe aortic stenosis (AS) (1). The pathobiology of AVS and AS shares similarities with atherosclerosis involving lipid accumulation, inflammation, and calcification (2). The link between lipid, inflammation, and calcification in calcific AoV disease and the pathological similarities with atherosclerosis led to the hypothesis that statins might be beneficial in patients with AS. Some retrospective studies showed lipid-lowering therapies could prevent the progression to overt AS (3–5). However, prospective studies have demonstrated a failure to attenuate the progression of AS in statin-treated patients (6–8). The most plausible explanation for this inconsistent results is that whilst lipid deposition may play a pivotal role in the initiation phase, it has little effect in the advanced phase when fibrosis and calcification are the dominant pathological processes.

Hence, the independent contribution of atherosclerotic risk factors to disease progression at various stages of calcific AV disease remains unclear. In response, this study sought to determine the impact of contributing risk factors on the progression of patients with AVS and mild to moderate AS.

## Methods

### Study population

**Figure 1:**
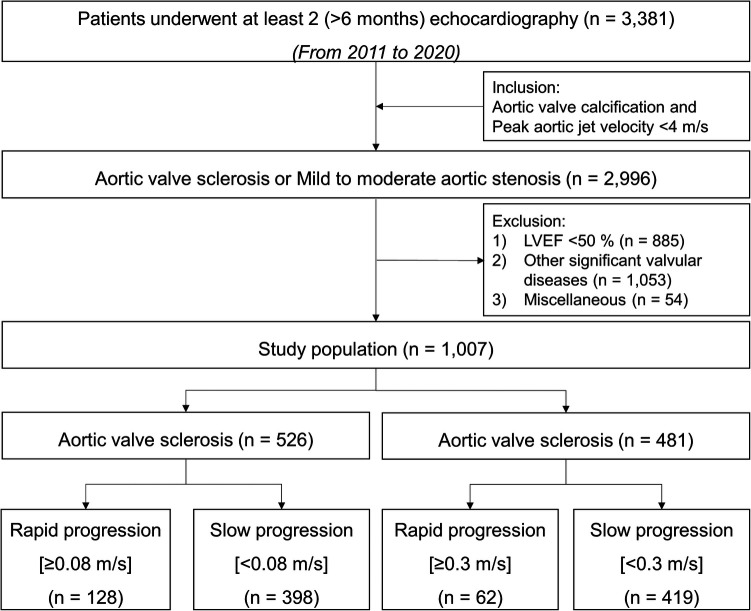
Flow diagram.

We retrospectively included 1,007 patients with AVS (irregular leaflet thickening, focally increased echogenicity) revealed by 2-dimensional echocardiography and [peak aortic jet velocity (Vmax), <2 m/s] by Doppler echocardiography, mild AS [aortic valve area (AVA), 1.5–2.0 cm^2^; Vmax, 2.0–3.0 m/s], or moderate AS (AVA, 1.0–1.5 cm^2^; Vmax, 3.0–4.0 m/s) and subsequently selected patients who had undergone ≥2 echocardiography examinations at ≥6 months apart during 2011–2020. Patients with other significant valvular diseases, left ventricular dysfunction [left ventricular ejection fraction (LVEF) < 50%], congenital heart diseases, cardiomyopathy, a permanent pacemaker, or a history of cardiac surgery were excluded. Flow diagram was presented in Figure 1.

The progression rates of AVS, mild AS, and moderate AS during a median follow-up period of 2.3 (interquartile range, 1.3–3.5) years were 0.01 (−0.10 to 0.08), 0.06 (0.00–0.16), and 0.17 (0.04–0.28) m/s/yr, respectively (Supplementary Figure S1). Patients were identified as a rapid progression group if the annualized difference in Vmax between two echocardiographic examinations was >0.08 m/s/yr (highest quartile) in AVS and >0.3 m/s/yr in AS, respectively.

The study protocol was approved by the institutional review board of a single center (KNUH IRB File No. 2022-02-010), and the need for informed consent was waived because of the retrospective nature of the study.

### Clinical data

Clinical data, including the medical history and presence of risk factors, were obtained by a complete review of patient medical records. The presence of dyslipidemia was defined by a total cholesterol >200 mg/dl or use of lipid-lowering therapy; diabetes mellitus was defined by a fasting plasma glucose >126 mg/dl, plasma glucose level >200 mg/dl tested twice, or use of anti-diabetic medication; hypertension was defined by blood pressure ≥140/90 mmHg at office or use of anti-hypertensive medication; and coronary artery disease (CAD) was defined by previously documented myocardial infarction or coronary artery stenosis with a lumen diameter >50% on angiography.

### Echocardiography

Comprehensive transthoracic echocardiography was performed using commercially available equipment (Vivid E9 from GE Healthcare, Milwaukee, WI, USA or Acuson SC2000 from Siemens Medical Solutions, Mountain View, CA, USA). Standard M-mode, 2-dimensional, and color Doppler imaging were performed in parasternal, suprasternal, substernal, and apical views with positional adjustment of the patient. The first and last echocardiograms collected during the study period were used to evaluate echocardiographic changes. Anatomic measurements were performed according to the American Society of Echocardiography and the European Association of Cardiovascular Imaging (9).

### Statistical analysis

Continuous variables were tested for normality using the Shapiro–Wilk test. Results were expressed as mean ± standard deviation or median (25th–75th percentile) and compared with Student's *t* test or the Wilcoxon rank-sum test between patients with rapid versus slow progression in the AVS and mild to moderate AS groups. Categorical variables are presented as percentages and were compared with the Chi-square test or Fisher's exact test, as appropriate.

Multivariable logistic regression analyses were performed to assess the factors associated with rapid progression or progression to severe AS in AVS and mild to moderate AS patients, after adjusting for clinically relevant variables and variables with *p* < 0.20 in univariate analysis and carefully avoiding collinearity. The variables adjusted were age, sex, body mass index, smoking status, hypertension, diabetes, dyslipidemia, CAD, C-reactive protein (CRP) level, and LVEF.

*p* < 0.05 was considered statistically significant. Statistical analyses were performed using the R statistical software program (version 4.2.1; R Foundation for Statistical Computing, Vienna, Austria) and SPSS software version 25.0 (IBM Corp., Armonk, NY, USA).

## Results

### Patient characteristics

Baseline characteristics are listed in Table 1. Among the 526 AVS patients (128 with rapid progression and 398 with slow progression), those with rapid progression were older (74 ± 8 vs. 69 ± 12 years, *p* = <0.001). In the rapid-progression group of AVS patients, the Vmax was 1.74 ± 0.15 m/s, the AVA was 1.83 ± 0.57 cm^2^, and the rate of progression was 0.19 (range, 0.13–0.32) m/s/yr. Co-morbidities and laboratory findings were comparable between the groups (all *p* > 0.08).

**Table 1:** Baseline characteristics.

	Aortic valve sclerosis (*n* = 526)			Mild to moderate AS (*n* = 481)		
	Rapid progression (median f/u: 1.5 yr)	Slow progression (median f/u: 2.1 yr)	*p* Value	Rapid progression (median f/u: 1.4 yr)	Slow progression (median f/u: 2.9 yr)	*p* Value
Clinical data						
Age, years	74 ± 8	69 ± 12	**< 0.001**	77 ± 11	75 ± 9	0.175
Male	50 (39)	162 (41)	0.742	33 (53)	151 (36)	**0**.**009**
Body mass index, kg/m^2^	24.4 ± 4.1	25.1 ± 4.1	0.086	24.7 ± 4.0	24.4 ± 3.8	0.489
SBP, mmHg	129 ± 19	129 ± 17	0.841	134 ± 23	132 ± 20	0.471
DBP, mmHg	76 ± 10	77 ± 11	0.418	76 ± 11	76 ± 12	0.889
Smoking ever	10 (8)	41 (10)	0.408	9 (15)	60 (14)	0.967
Hypertension	100 (78)	301 (76)	0.564	47 (76)	353 (84)	0.097
Diabetes	42 (33)	134 (34)	0.858	22 (36)	149 (36)	0.991
Dyslipidemia	69 (54)	240 (60)	0.201	40 (65)	272 (65)	0.951
Coronary artery disease	48 (38)	141 (35)	0.671	11 (18)	84 (20)	0.670
Cerebrovascular accident	29 (23)	77 (19)	0.417	17 (27)	109 (26)	0.814
Statin use	62 (48)	197 (50)	0.835	45 (73)	279 (67)	0.348
RAS blocker	70 (55)	215 (54)	0.722	39 (63)	288 (69)	0.358
Beta blocker	44 (34)	123 (31)	0.202	28 (45)	165 (39)	0.386
Calcium channel blocker	50 (39)	139 (35)	0.108	37 (60)	214 (51)	0.206
Diuretics	51 (40)	131 (33)	**0**.**005**	37 (60)	229 (55)	0.458
Laboratory data						
Hemoglobin, g/dL	12.4 ± 2.2	12.4 ± 2.2	0.965	12.1 ± 1.9	12.0 ± 2.2	0.597
Creatinine, mg/dL	0.9 (0.7, 1.1)	0.8 (0.7, 1.1)	0.713	0.9 (0.7, 1.2)	0.8 (0.6, 1.1)	**0**.**011**
Uric acid, mg/dl	5.3 ± 2.0	5.5 ± 2.9	0.490	5.4 ± 2.1	5.6 ± 4.5	0.749
Glucose, mg/dl	142 ± 72	136 ± 60	0.389	129 ± 41	129 ± 51	0.998
HbA1c, %	6.9 ± 1.9	6.6 ± 1.2	0.297	6.7 ± 1.6	6.5 ± 1.3	0.289
Calcium, mg/dl	8.9 ± 0.6	9.2 ± 4.9	0.477	8.9 ± 0.6	9.1 ± 4.4	0.684
CRP, mg/dl	0.27 (0.09, 1.60)	0.23 (0.05, 1.16)	0.255	0.37 (0.06, 1.52)	0.25 (0.05, 1.84)	0.135
Total cholesterol, mg/dl	167 ± 44	164 ± 45	0.578	164 ± 45	161 ± 41	0.628
LDL-C, mg/dl	102 ± 38	96 ± 39	0.143	103 ± 38	97 ± 38	0.262
Follow-up LDL, mg/dl	78 ± 35	80 ± 33	0.544	82 ± 34	81 ± 33	0.870
Echocardiographic data						
LVEDD, mm	48.3 ± 6.7	48.8 ± 6.2	0.509	49.1 ± 6.6	47.9 ± 5.7	0.141
LVESD, mm	31.6 ± 7.5	31.6 ± 7.0	0.935	32.9 ± 7.5	30.4 ± 5.4	**0**.**013**
LVEF, %	61.6 ± 11.0	61.8 ± 10.9	0.847	61.2 ± 11.8	65.0 ± 8.5	**0**.**019**
LAVI, ml/m^2^	45.8 ± 19.1	45.8 ± 23.3	0.991	52.3 ± 23.6	48.3 ± 23.0	0.211
E velocity, m/s	0.69 ± 0.25	0.70 ± 0.25	0.612	0.72 ± 0.23	0.69 ± 0.26	0.429
A velocity, m/s	0.88 ± 0.19	0.87 ± 0.23	0.468	0.98 ± 0.22	0.93 ± 0.22	0.144
E/e' ratio	14.1 ± 6.2	13.3 ± 6.0	0.237	15.9 ± 7.1	13.8 ± 6.0	**0**.**016**
RVSP, mmHg	32.5 ± 11.5	32.3 ± 12.0	0.904	33.5 ± 12.3	31.9 ± 9.8	0.332
Peak aortic jet velocity, m/s	1.74 ± 0.15	1.73 ± 0.14	0.255	2.70 ± 0.52	2.49 ± 0.45	**< 0.001**
Aortic valve area, cm^2^	1.83 ± 0.57	1.91 ± 0.62	0.243	1.53 ± 0.30	1.71 ± 0.33	**< 0.001**
Rate of progression, m/s/yr	0.19 (0.13, 0.32)	−0.05 (–0.13, 0.01)	**< 0.001**	0.46 (0.35, 0.73)	0.05 (0.00, 0.13)	**< 0.001**

Values are presented as mean ± standard deviation or number (%) or median (interquartile range).

A, late diastolic mitral inflow velocity; AS, aortic stenosis; CRP, C-reactive protein; DBP, diastolic blood pressure; E, early diastolic mitral inflow velocity; E/e’, Early diastolic velocity of the mitral annulus; f/u, follow-up; HbA1c, Hemoglobin A1c; LAVI, left atrial volume index; LVEDD, left ventricular end-diastolic dimension; LDL-C, low-density lipoprotein cholesterol; LVEF, left ventricular ejection fraction; LVESD, left ventricular end-systolic dimension; RAS, renin-angiotensin system; RVSP, right ventricular systolic pressure; SBP, systolic blood pressure.

Bold formatting of values indicates the presence of statistical significance (*p* < 0.05).

Among the 481 mild to moderate AS patients (62 with rapid progression and 419 with slow progression), there were significant differences between the rapid- and slow-progression groups in terms of male sex, creatinine, left ventricular end-systolic dimension (LVESD), LVEF, and E/e’. In the rapid-progression group of mild to moderate AS patients, the Vmax was 2.70 ± 0.52 m/s, the AVA was 1.53 ± 0.30 cm^2^, and the rate of progression was 0.46 (range, 0.35–0.73) m/s/yr.

### Atherosclerotic risk factors for the progression of AVS

**Figure 2:**
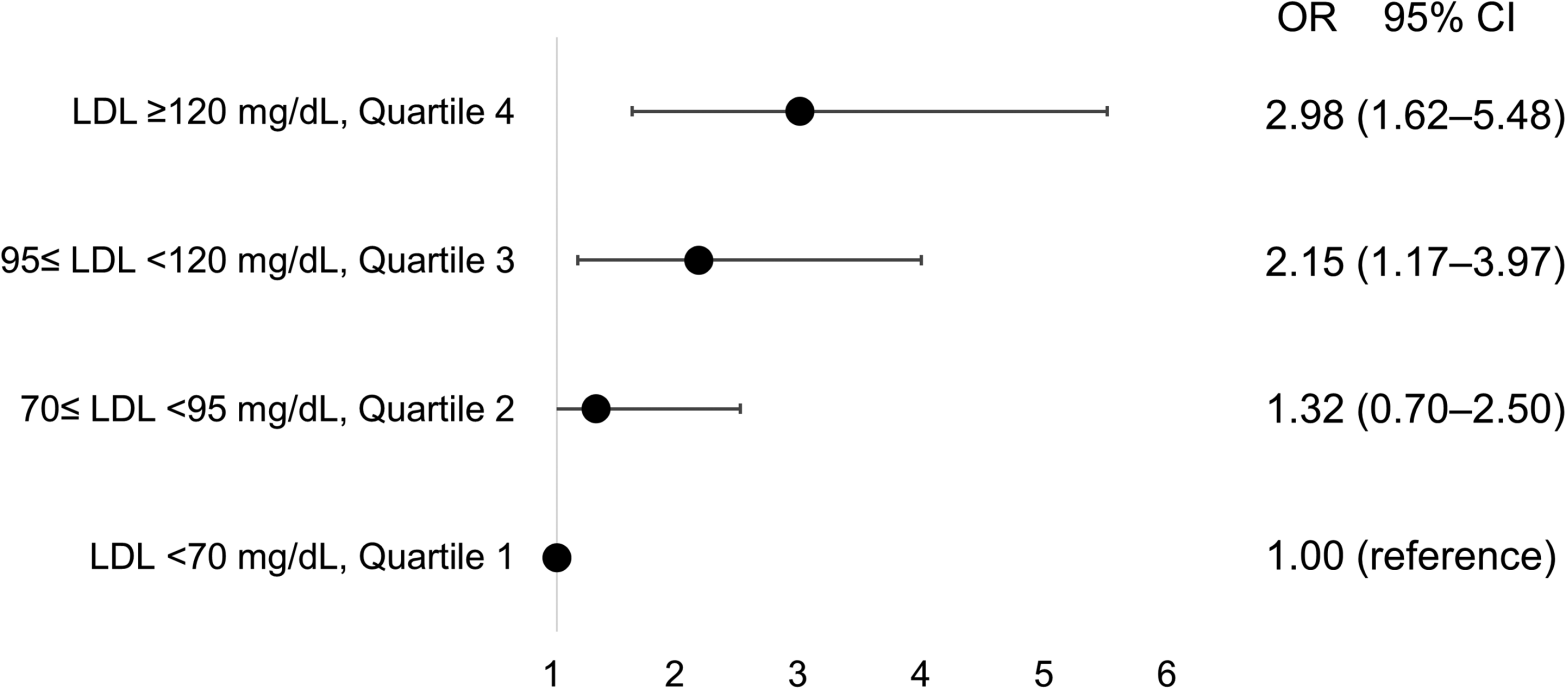
Incremental risk of progression in aortic valve sclerosis according to concentrations of low-density lipoprotein. CI, confidence interval; LDL, low-density lipoprotein; OR, odds ratio.

In univariate analysis, age, body mass index, and LDL-C were significant (all *p* < 0.20). After adjustment for smoking, hypertension, diabetes, dyslipidemia, CAD and CRP level, LDL-C level (odds ratio [OR] 1.22/per 25 mg/dl higher LDL-C, 95% confidence interval [CI] 1.05–1.43) and age (OR 1.04/per 1 year higher age, 95% CI 1.02–1.07) were significantly associated with rapid disease progression in AVS patients (Table 2). The impact of LDL-C on AVS progression was attenuated by statin use, but showed consistent results regardless of statin (Supplementary Figure S2). Compared to patients with LDL-C < 70 mg/dl, the adjusted OR for rapid progression was 1.32 (95% CI 0.70–2.50) for those with LDL-C level 70–95 mg/dl, 2.15 (95% CI 1.17–3.97) for those with LDL-C level 95–120 mg/dl, and 2.98 (95% CI 1.62–5.48) for those with LDL-C level ≥120 mg/dl (Figure 2).

**Table 2:** Unadjusted and adjusted logistic regression analyses for rapid progression in patients with aortic valve sclerosis.

	Univariable		Multivariable	
	OR (95% CI)	*p* value	OR (95% CI)	*p* value
Age, per 1 year increase	1.04 (1.02–1.07)	< 0.001*	1.04 (1.02–1.07)	0.001
Male gender	0.93 (0.62–1.40)	0.742		
Body mass index, kg/m^2^	0.96 (0.91–1.01)	0.086*		
Smoking ever	0.74 (0.36–1.52)	0.409		
Hypertension	1.15 (0.71–1.86)	0.564		
Diabetes	0.96 (0.63–1.47)	0.858		
Dyslipidemia	0.77 (0.52–1.15)	0.202		
Coronary artery disease	1.09 (0.72–1.65)	0.671		
CRP, mg/dl	1.03 (0.99–1.07)	0.207		
LDL-C, per 25 mg/dl increase	1.10 (0.97–1.26)	0.145*	1.22 (1.05–1.43)	0.011

CRP, C-reactive protein; LDL-C, low-density lipoprotein cholesterol.

* *p* value indicates the presence of statistical significance (*p* < 0.20).

### Vmax but not atherosclerotic risk factors for the progression of mild to moderate AS

**Figure 3:**
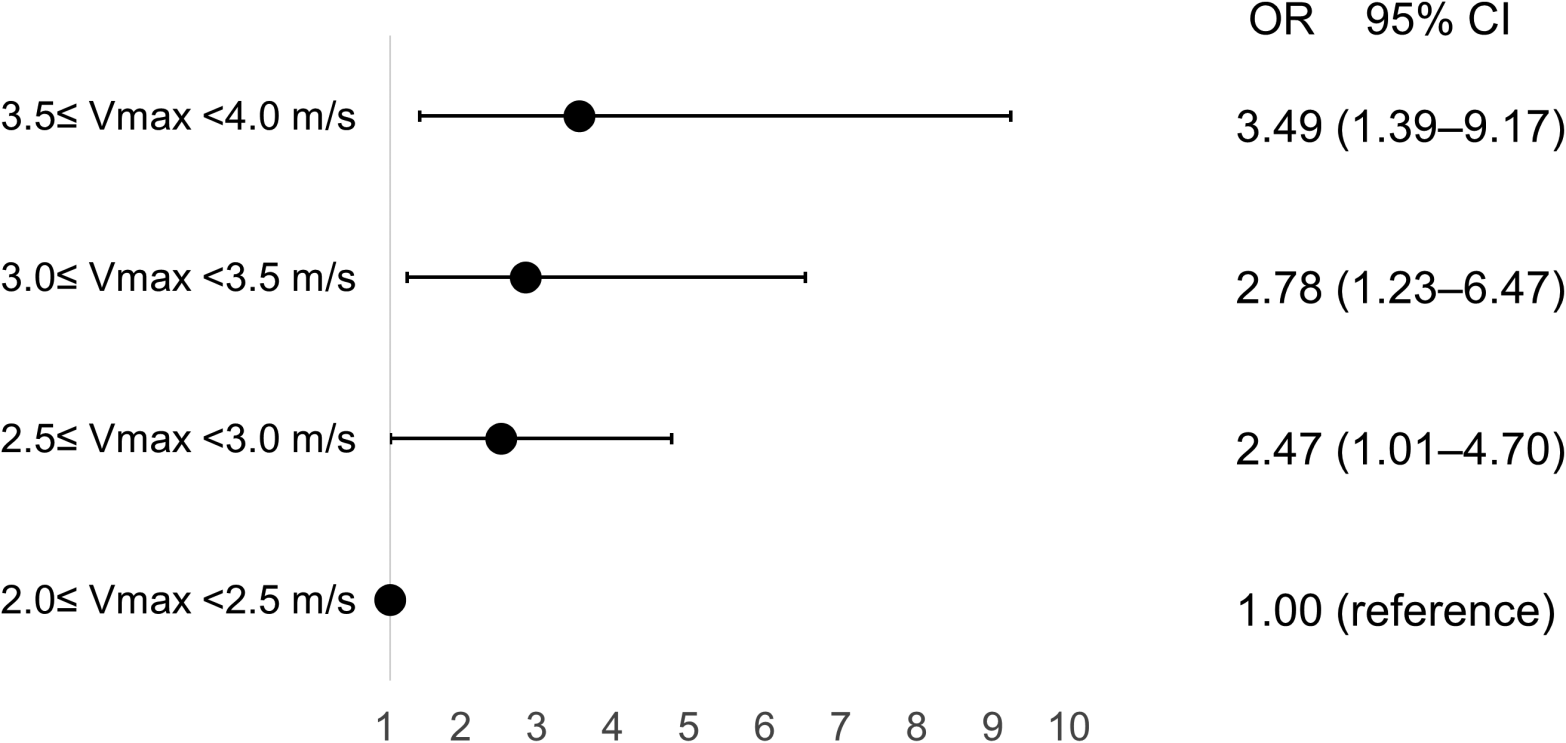
Incremental risk of progression in aortic valve stenosis according to baseline peak aortic jet velocity. CI, confidence interval; OR, odds ratio; Vmax, peak aortic jet velocity.

Atherosclerotic risk factors were not associated with rapid disease progression among mild to moderate AS patients; however, baseline Vmax (OR 1.79/per 0.5 m/s higher Vmax, 95% CI 1.18–2.70) and E/e′ (OR 1.08, 95% CI 1.01–1.15) were significantly associated with rapid disease progression in patients with mild to moderate AS (Table 3). Compared to patients with Vmax 2.0–2.5 m/s, the adjusted OR for rapid progression was 2.47 (95% CI 1.01–4.70) for those with Vmax 2.5–3.0 m/s, 2.78 (95% CI 1.23–6.47) for those with Vmax 3.0–3.5 m/s, and 3.49 (95% CI 1.39–9.17) for those with Vmax 3.5–4.0 m/s (Figure 3). Initial and follow-up AoV mean pressure gradient and AVA were also presented in Supplementary Figure S3.

**Table 3:** Unadjusted and adjusted logistic regression analyses for rapid progression in patients with mild to moderate aortic stenosis.

	Univariable		Multivariable	
	OR (95% CI)	*p* value	OR (95% CI)	*p* value
Follow-up durations, year	0.57 (0.46–0.70)	< 0.001*	0.59 (0.45–0.76)	< 0.001
Age, year	1.02 (0.99–1.06)	0.175*		
Male gender	2.02 (1.18–3.46)	0.010*		
Body mass index, kg/m^2^	1.03 (0.96–1.10)	0.488		
Smoking ever	1.02 (0.48–2.17)	0.967		
Hypertension	0.59 (0.31–1.11)	0.100*		
Diabetes	1.00 (0.57–1.74)	0.991		
Dyslipidemia	0.98 (0.56–1.72)	0.951		
Coronary artery disease	0.86 (0.43–1.72)	0.671		
Creatinine, mg/dl	0.81 (0.56–1.16)	0.254		
CRP, mg/dl	0.97 (0.90–1.04)	0.315		
LDL-C, mg/dl	1.00 (0.99–1.01)	0.262		
LVEDD, mm	1.03 (0.99–1.08)	0.141*		
LVESD, mm	1.07 (1.03–1.11)	0.002*		
LVEF, %	0.97 (0.94–0.99)	0.004*		
A velocity, m/s	2.78 (0.71–10.8)	0.144*		
E/e’ ratio	1.05 (1.01–1.09)	0.018*	1.08 (1.01–1.15)	0.026
Peak aortic jet velocity, per 0.5 m/s increase	1.57 (1.28–1.93)	< 0.001*	1.79 (1.18–2.70)	0.006

A, late diastolic mitral inflow velocity; CRP, C-reactive protein; E/e′, Early diastolic velocity of the mitral annulus; LDL-C, low-density lipoprotein cholesterol; LVEDD, left ventricular end-diastolic dimension; LVEF, left ventricular ejection fraction; LVESD, left ventricular end-systolic dimension; RVSP, right ventricular systolic pressure.

* *p* value indicates the presence of statistical significance (*p* < 0.20).

### Contributing factors associated with progression to severe AS

During a median follow-up period of 2.3 years, no AVS patients progressed to severe AS, while 12 (3.0%) patients progressed from mild to severe AS and 31 (40%) patients progressed from moderate to severe AS (Supplementary Figure S4). Among all patients with calcific AV disease, LDL-C level (OR 1.23/per 25 mg/dl higher LDL-C, 95% CI 1.02–1.50), baseline Vmax (OR 6.38/per 0.5 m/s higher Vmax, 95% CI 4.12–9.89) were significantly associated with progression to severe AS (Table 4).

**Table 4:** Unadjusted and adjusted logistic regression analyses for progression to severe aortic stenosis in total patients with calcific aortic valve disease.

	Univariable		Multivariable	
	OR (95% CI)	*p* value	OR (95% CI)	*p* value
Follow-up durations, year	1.67 (1.45–1.91)	< 0.001*	1.63 (1.34–1.98)	< 0.001
Age, year	1.03 (1.00–1.07)	0.074*		
Male gender	1.50 (0.81–2.76)	0.194*		
Body mass index, kg/m^2^	0.99 (0.93–1.06)	0.764		
Smoking ever	2.03 (0.95–4.35)	0.067*		
Hypertension	1.13 (0.52–2.48)	0.758		
Diabetes	1.13 (0.60–2.13)	0.698		
Dyslipidemia	1.46 (0.75–2.83)	0.267		
Coronary artery disease	0.48 (0.21–1.10)	0.082*		
CRP, mg/dl	0.96 (0.87–1.05)	0.346		
LDL-C, per 25 mg/dl increase	1.21 (1.01–1.45)	0.034*	1.23 (1.02–1.50)	0.034
Peak aortic jet velocity, per 0.5 m/s increase	5.02 (3.62–6.95)	< 0.001*	6.38 (4.12–9.89)	< 0.001

CRP, C-reactive protein; LDL, low-density lipoprotein cholesterol.

* *p* value indicates the presence of statistical significance (*p* < 0.20).

## Discussion

**Figure 4:**
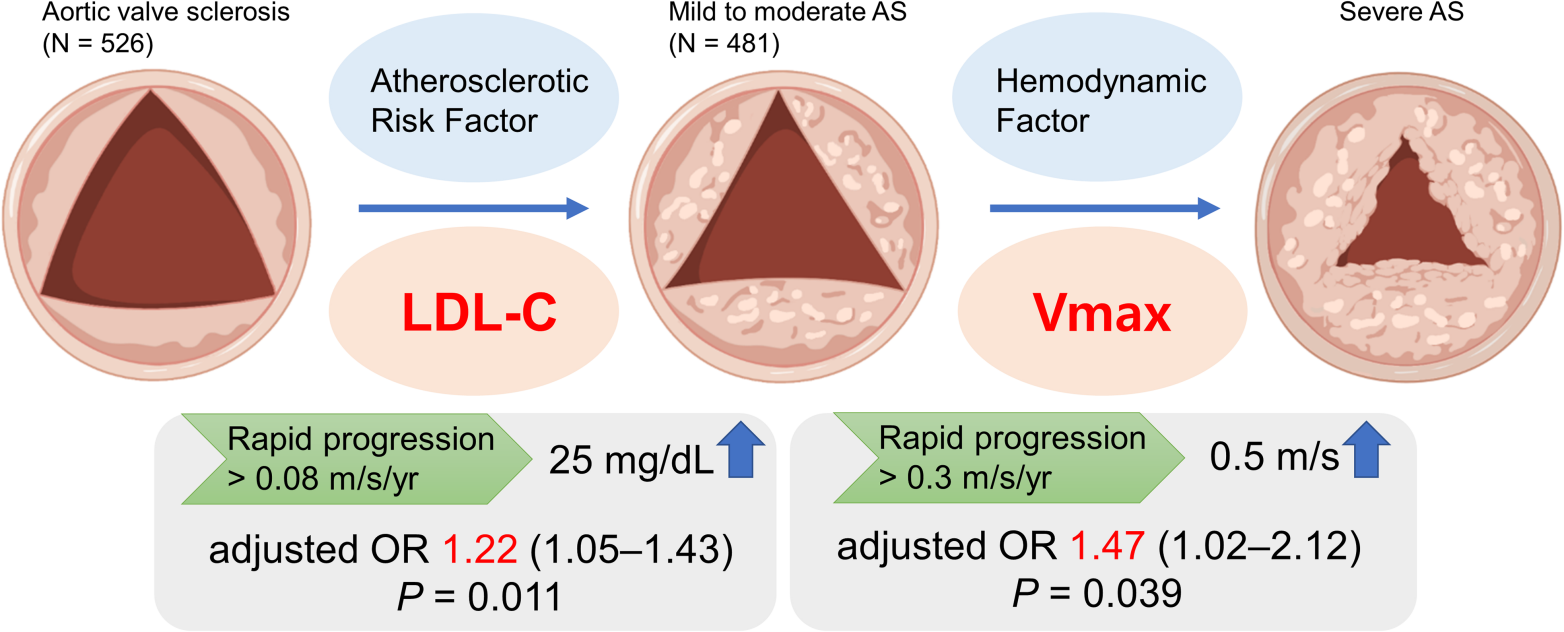
In a single center registry, low-density lipoprotein cholesterol (LDL-C) among atherosclerotic risk factors was associated with the rapid progression of aortic valve sclerosis (AVS) in multivariable analysis. In contrast, progression of mild to moderate aortic stenosis (AS) was associated with baseline peak aortic jet velocity (Vmax). AS, aortic stenosis; LDL-C, low-density lipoprotein cholesterol; Vmax, peak aortic jet velocity.

The main findings of this study are that: (1) Atherosclerotic risk factors were significantly associated with the rapid progression in AVS patients and LDL-C showed a markedly incremental risk of AVS progression; (2) baseline Vmax, not atherosclerotic risk factors, was associated with the rapid progression in patients with mild to moderate AS; (3) LDL-C and baseline Vmax were independently associated with progression to severe AS in patients with calcific AoV disease (Figure 4).

### Atherosclerotic risk factors for AVS

Some studies have reported the frequent coexistence of either AVS or AS in patients with underlying CAD (10). However, research to date has not been able to prove causality despite the frequent coexistence of these entities. Multicenter study showed that AVS was strongly associated with the presence and degree of CAD independently of clinical risk factors (11). A prospective study found a higher incidence of cardiovascular events and worse survival in AVS patient, but after adjustment such as CAD and CRP, no statistically significant differences were found (12). Other prospective study demonstrated a higher risk of myocardial infarction and cardiovascular mortality in subjects with AVS and no known CAD, after adjustment for traditional cardiac risk factors (13). The association between AVS and CAD warrants further research. The initial lesions in both AVS and CAD involve lipid deposition and focal sclerosis (2). The early phase of the disease, observed in patients with AVS, is characterized by prominent accumulation of LDL-C and lipoprotein(a) [Lp(a)] (14, 15). The current study demonstrates the incremental risk of LDL-C in the rapid progression of AVS. This result reveals LDL-C is important in the initiating step in the development of AVS. The extracellular lipid infiltration causes LDL oxidization, stimulates inflammatory process, and finally calcification (16). In this study, the association between LDL-C and the rapid progression of AVS is weak in univariable analysis (*p* > 0.10) and significant in multivariable anaylsis, therefore, it is difficult to reveal clearly that LDL-C affects AVS progression because of a confounder's effect. In addition, there is no significant difference on follow-up LDL-C between rapid and slow progression group in AVS patients, and this suggests the existence of other contributing factors. Well-controlled research about the impact of LDL-C on the progression of AVS is needed. Some patients with slow progression had a decline in transaortic velocity. First, it is possible that hemodynamic progression has not been established in AVS stages. Second, there is more difference in the measurement of Vmax in AVS than in AS. This is due to a suboptimal Doppler study with a nonparallel intercept angle. The principle that lower LDL-C is better in cardiovascular disease (17) may have more evidence for application in patients with AVS.

### Hemodynamic factors but not atherosclerotic risk factors for progressive AS

In this study, the role of atherosclerotic risk factors is not proven in the progression of mild to moderate AS. The study shows that higher baseline Vmax is associated with the rapid progression of AS. Upon mild valve obstruction, disease progression dictated neither by inflammation nor by lipid deposition, but rather by increasing hemodynamic severity (18). The stages of AS are characterized by fibrosis and accelerated calcification, leading to valvular dysfunction and changes in mechanical stress and flow (19). In addition to hemodynamic progression in the advanced stages of calcific AS, it has been speculated that hypertension and the increased stiffness of the aortic root that occurs with ageing may also cause abnormally high mechanical stress in the valve (20, 21). An unmet need exists to develop new pharmacological treatment strategies delaying calcific AS progression.

### The association between LDL-C and Vmax in the progression of calcific AoV disease

A gradual progression of calcific AV disease may ultimately to severe AS, which eventually leads to ventricular remodeling and hemodynamic compromise with a high morbidity and mortality if not treated (22). In this study, LDL-C and baseline Vmax are independently associated with progression to severe AS in total patients with calcific AV disease. Although the early stage of calcific AV disease is mainly mediated by lipid deposition and inflammation, the role of hemodynamic progression is more prominent in the later stage (23). In line, recent study has reported that Lp(a) is associated with new-onset AV calcium but not with AV calcium progression (24). Although statin attenuated the impact of LDL-C on AVS progression in subgroup analysis, it was not statistically significant. More research regarding the pathophysiology of calcific AoV disease continuum and novel targets holding potential for the progression is needed.

### Study limitations

This study has several limitations. First, the retrospective nature of the study does not exclude other potential confounding variables not included in the analysis could have affected the results. Second, the study has a relatively short-term period to fully observe the progression of AVS. Third, we did not measure Lp(a), a biomarker for AS progression. However, we do not think the check for Lp(a) is routine in the current clinical practice. Fourth, only patients who underwent follow-up echocardiograhy were included in this study, therefore, selection bias might also affect the results. Fifth, this study did not show clinical events such as aortic valve intervention or mortality. However, observation of progression to severe AS is important due to its high morbidity. Finally, this study limits the participants to a single center and a single ethnicity. Hence, our findings should be expanded and further verified in well-controlled prospective studies.

In conclusion, atherosclerotic risk factors such as LDL-C were significantly associated with the rapid progression in AVS and baseline Vmax was important in the stage of mild to moderate AS. These findings provide insights for future research to identify novel therapeutic targets which alters the course of calcific AoV disease.

## Data Availability

The raw data supporting the conclusions of this article will be made available by the authors, without undue reservation.

## Supplementary material

Supplementary Figure S1. The rate of progression according to the grade of calcific aortic valve disease. **p*-value determined by one-way ANOVA *Abbreviations:* AS, aortic valve stenosis; AVS, aortic valve sclerosis.

Supplementary Figure S2. Subgroup analysis for the impact of LDL-C on AVS progression according to statin use. *Abbreviations:* AVS, aortic valve sclerosis; LDL-C, low-density lipoprotein cholesterol.

Supplementary Figure S3. Initial and follow-up AoV mean pressure gradient (A) and AVA (B). *Abbreviations:* AoV, aortic valve; AVA, aortic valve area.

Supplementary Figure S4. The ratio of progression to severe aortic valve stenosis according to the grade of calcific aortic valve disease. **p*-value determined by the chi-square test. *Abbreviations:* AS, aortic valve stenosis; AVS, aortic valve sclerosis.
