# Supplementary figures and images for: Impact of low-density lipoprotein cholesterol on progression of aortic valve sclerosis and stenosis
Source: Front Cardiovasc Med. 2023 Jul 17;10:1171703. doi: 10.3389/fcvm.2023.1171703 (PMC10390070; doi:10.3389/fcvm.2023.1171703)

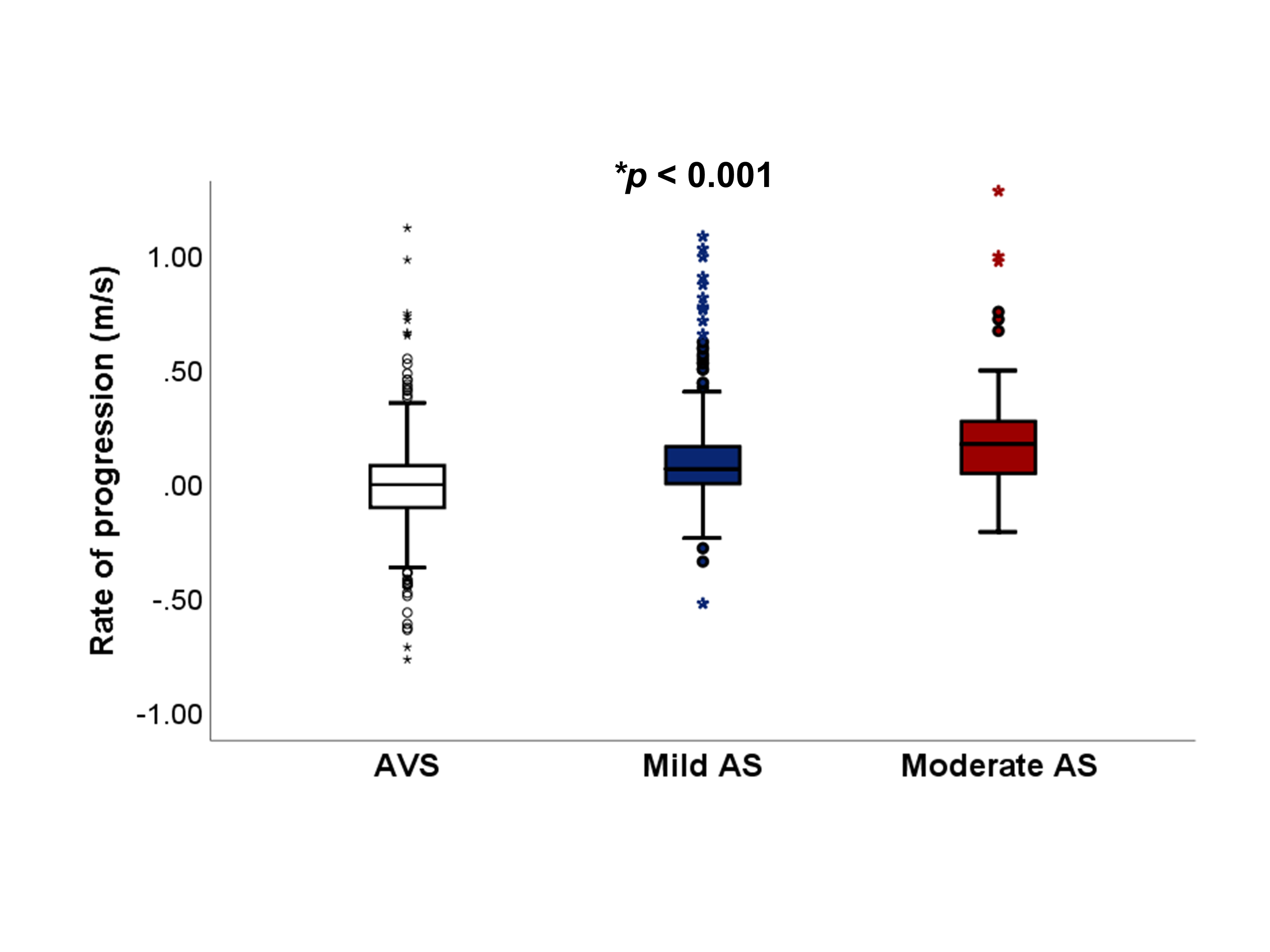

Supplement: Supplementary Figure S1. — The rate of progression according to the grade of calcific aortic valve disease. *p-value determined by one-way ANOVA Abbreviations: AS, aortic valve stenosis; AVS, aortic valve sclerosis. [file Image1.tif]

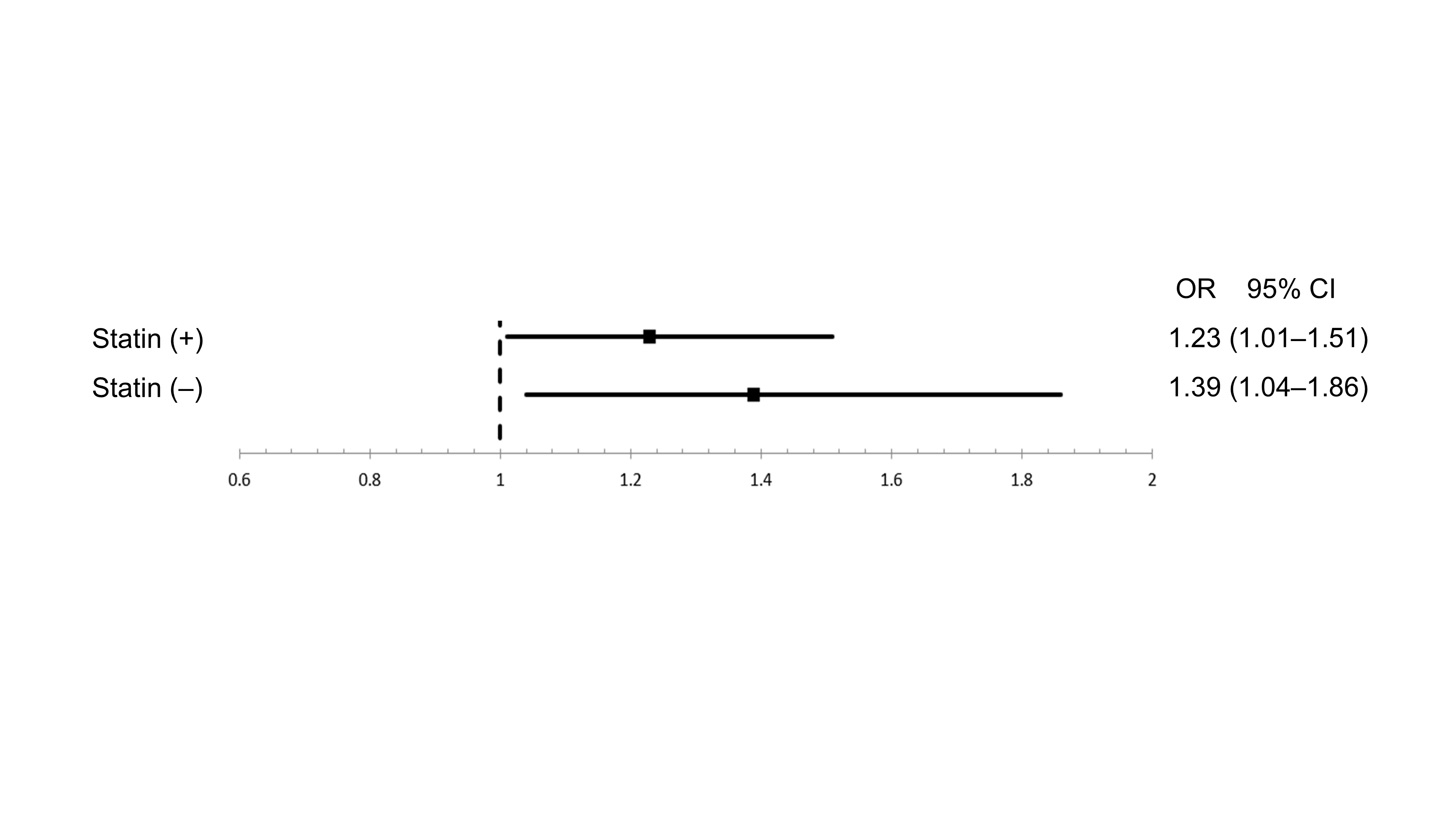

Supplement: Supplementary Figure S2. — Subgroup analysis for the impact of LDL-C on AVS progression according to statin use. Abbreviations: AVS, aortic valve sclerosis; LDL-C, low-density lipoprotein cholesterol. [file Image2.tif]

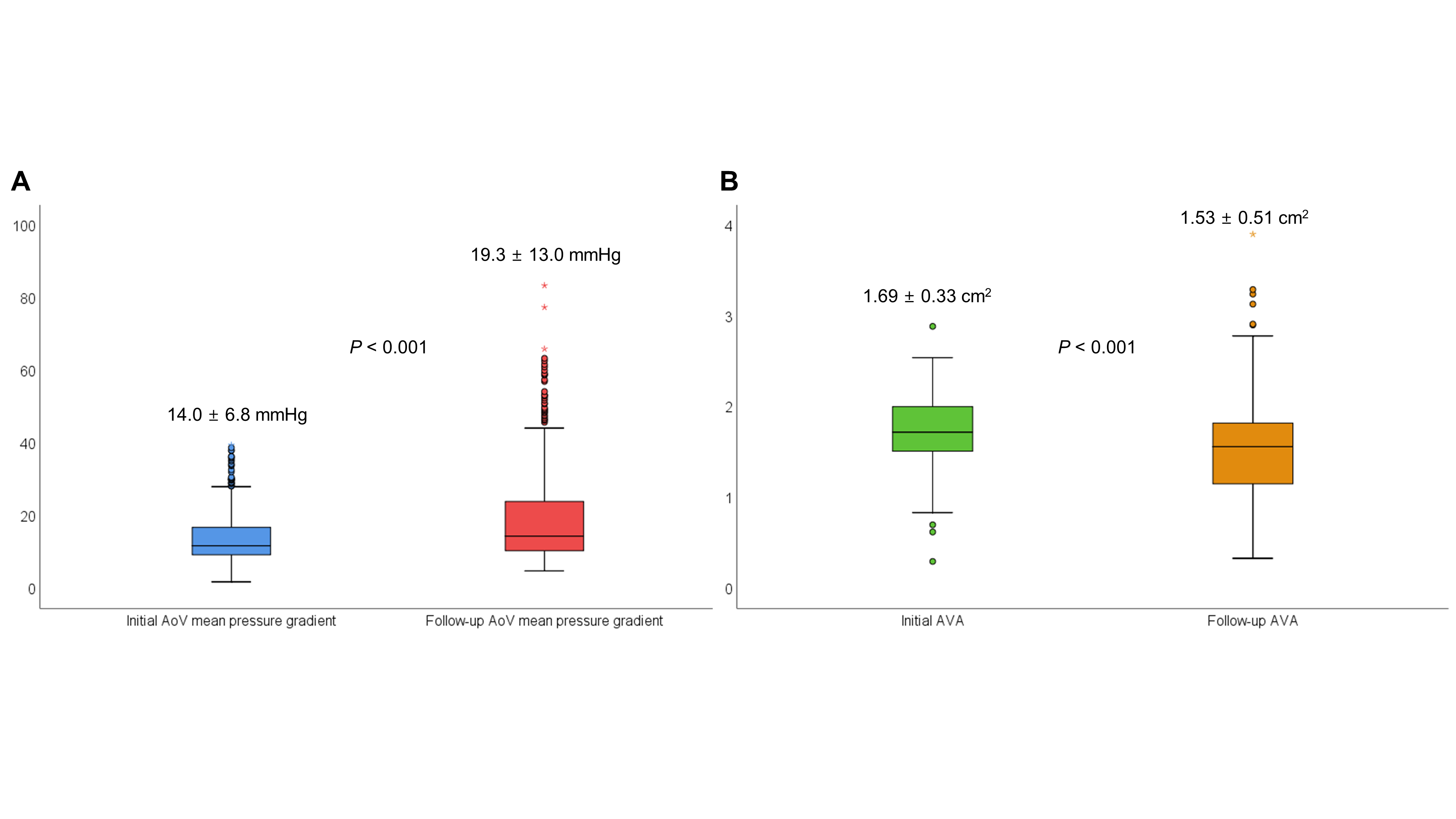

Supplement: Supplementary Figure S3. — Initial and follow-up AoV mean pressure gradient (A) and AVA (B). Abbreviations: AoV, aortic valve; AVA, aortic valve area. [file Image3.tif]

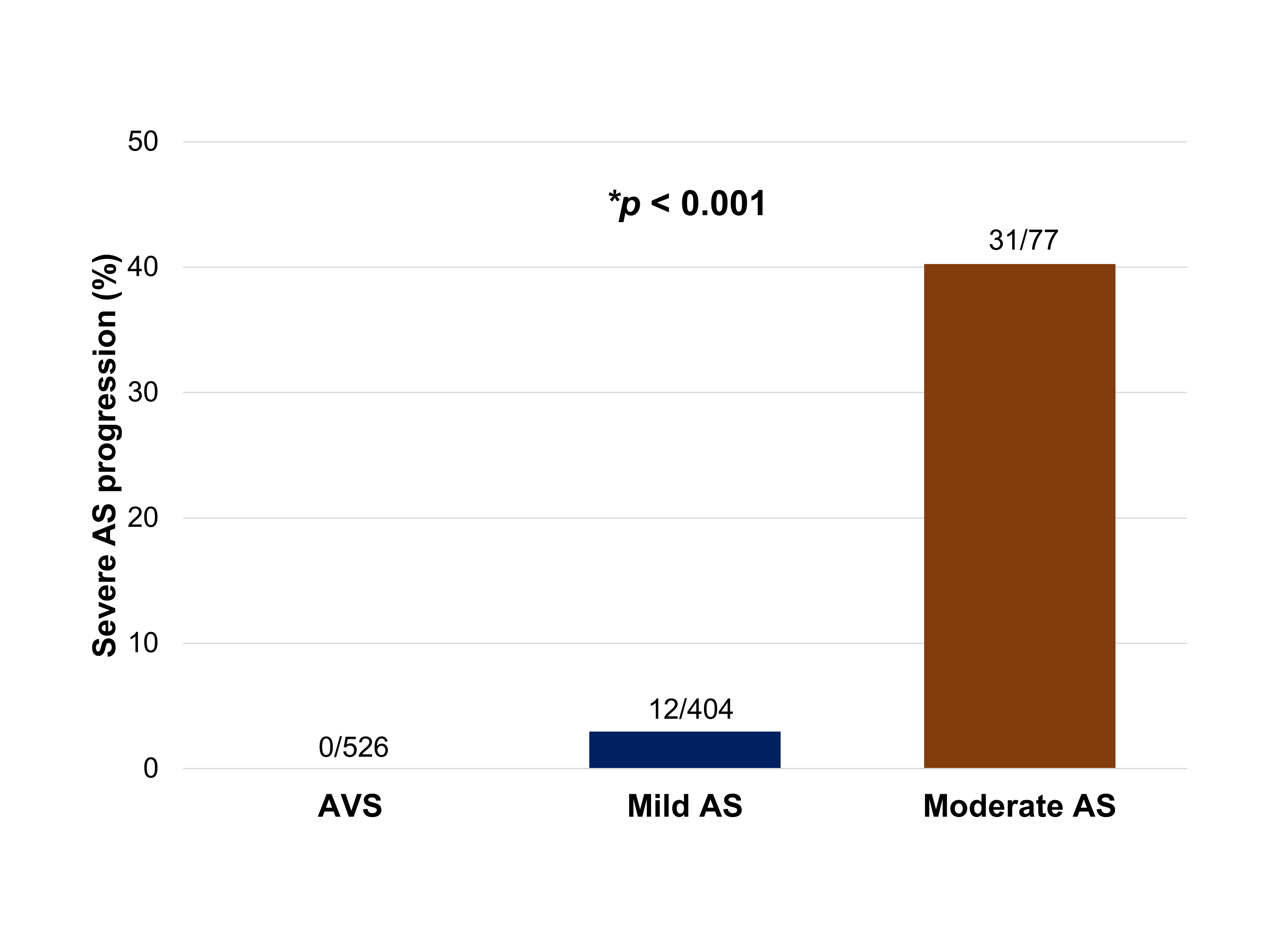

Supplement: Supplementary Figure S4. — The ratio of progression to severe aortic valve stenosis according to the grade of calcific aortic valve disease. *p-value determined by the chi-square test. Abbreviations: AS, aortic valve stenosis; AVS, aortic valve sclerosis. [file Image4.tif]
